# P25 Nowhere to hide: broad-spectrum antimicrobial guideline adherence in outpatients

**DOI:** 10.1093/jacamr/dlad143.029

**Published:** 2024-01-03

**Authors:** Shahzad Razaq, Waqar Raza, Abigail Jenkins

**Affiliations:** University Hospitals Birmingham NHS FT, UK; University Hospitals Birmingham NHS FT, UK; University Hospitals Birmingham NHS FT, UK

## Abstract

**Background:**

The NHS Acute Trusts Standard^1^ contract requires a 10% reduction in broad-spectrum antibiotics by 10% by March 2024 from a 2017 baseline. Co-amoxiclav is a broad-spectrum antibacterial that comprises 30% of broad-spectrum consumption at University Hospitals Birmingham. It is widely used across all specialities and is a perfect candidate to review to influence downward trend in consumption. Outpatient prescriptions do not receive the same level of scrutiny from pharmacy or antimicrobial stewardship team as inpatient prescriptions. If deviations from guidelines are identified, then this could be a potential area to address to reduce consumption.

**Methods:**

Sixty outpatient co-amoxiclav prescriptions were reviewed to assess appropriateness of prescribing during December 2022. Parameters assessed included the indication and duration of co-amoxiclav therapy and adherence to Trust antimicrobial guidelines. The speciality prescribing outpatient co-amoxiclav was also documented to identify whether prescribing trends could be identified.

**Results:**

Of 60 reviewed outpatient prescriptions written in December 2022, 54 were issued by acute medicine with other specialities including cardiothoracic, renal medicine, elderly care, ENT, urology and cardiology. All prescriptions had a documented indication and duration, however only three were adherent to Trust antimicrobial guidelines. The graph below represents the different indications co-amoxiclav was prescribed for to outpatients across all three hospital sites. Co-amoxiclav was most frequently prescribed for respiratory tract infections, followed by skin and then urinary tract infections.

**Conclusions:**

This audit has highlighted that outpatient co-amoxiclav is commonly prescribed inappropriately and does not comply with Trust antimicrobial guidelines. The speciality most frequently prescribing outpatient co-amoxiclav inappropriately was acute and emergency medicine. The lack of clarity regarding the infection indication is a potentially important factor in this setting, resulting in broad-spectrum antimicrobial use to cover multiple potential bacterial infections. A limitation of this audit is it was carried out over a short and busy winter period.
